# Low-temperature modification of Ba(BF_4_)_2_(H_2_O)_3_

**DOI:** 10.1107/S2414314623004881

**Published:** 2023-06-09

**Authors:** Evgeny Goreshnik, Andrii Vakulka, Gašper Tavčar

**Affiliations:** a Department of Inorganic Chemistry and Technology, Jožef Stefan Institute, Jamova 39 1000 Ljubljana, Slovenia; b Faculty of Mechanical Engineering, University of Ljubljana, Aškerčeva cesta 6, 1000 Ljubljana, Slovenia; Vienna University of Technology, Austria

**Keywords:** low-temperature modification, phase transition, barium tetra­fluorido­borate, crystal structure

## Abstract

The crystal structure of the low-temperature modification of Ba(BF_4_)_2_(H_2_O)_3_, barium bis(tetra­fluorido­borate) trihydrate, was determined at 150 K. In contrast to the room-temperature modification, which crystallizes in the space group *C*222_1_ [*a* = 7.1763 (6), *b* = 18.052 (2), *c* = 7.1631 (6) Å, *V* = 927.93 (15) Å^3^ at 300 K, *Z* = 4; Charkin *et al.* (2023 ▸). *J. Struct. Chem*. **64**, 253–261], the low-temperature phase is monoclinic, space group *P*2_1_ [*a* = 7.0550 (4), *b* = 7.1706 (3), *c* = 9.4182 (6) Å, *β* = 109.295 (7)^*o*^, *V* = 449.68 (5) Å^3^, *Z* = 2]. The structure of the low-temperature modification of Ba(BF_4_)_2_(H_2_O)_3_ features O—H⋯F and O—H⋯O hydrogen bonding between water mol­ecules and BF_4_^−^ anions. One of the coordinating water mol­ecules in the low-temperature modification is disordered over two sets of sites.

## Abstract 2

Ba(BF_4_)_2_(H_2_O)_3_, ortho­rhom­bic, space group *C*222_1_ at 300 K, becomes monoclinic at 280 K, space group *P*2_1_. The spread of Ba—F distances is significantly larger in the LT modification, and one of the water mol­ecules in the LT form exhibits positional disorder.

## Graphical abstract

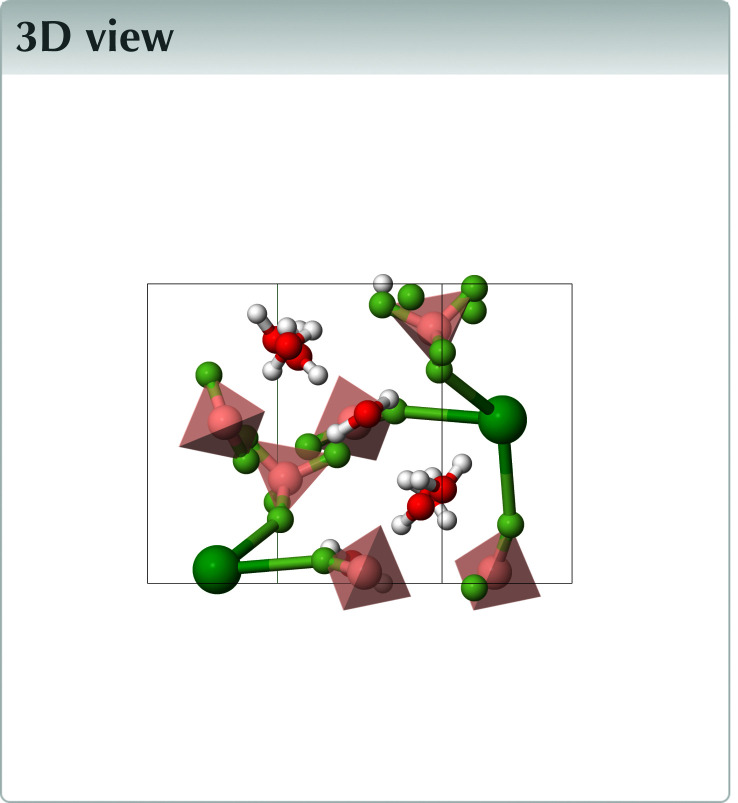

## Structure description

Recently, the ortho­rhom­bic crystal structure of the compound Ba(BF_4_)_2_(H_2_O)_3_ was reported on the basis of room-temperature (RT) single-crystal data in space group *C*222_1_ (Charkin *et al.*, 2023 ▸). The authors observed a phase transition at decreasing temperature but were unable to solve the crystal structure of the low-temperature (LT) modification. We have now succeeded in solving the crystal structure of LT-Ba(BF_4_)_2_(H_2_O)_3_.

**Figure 1:**
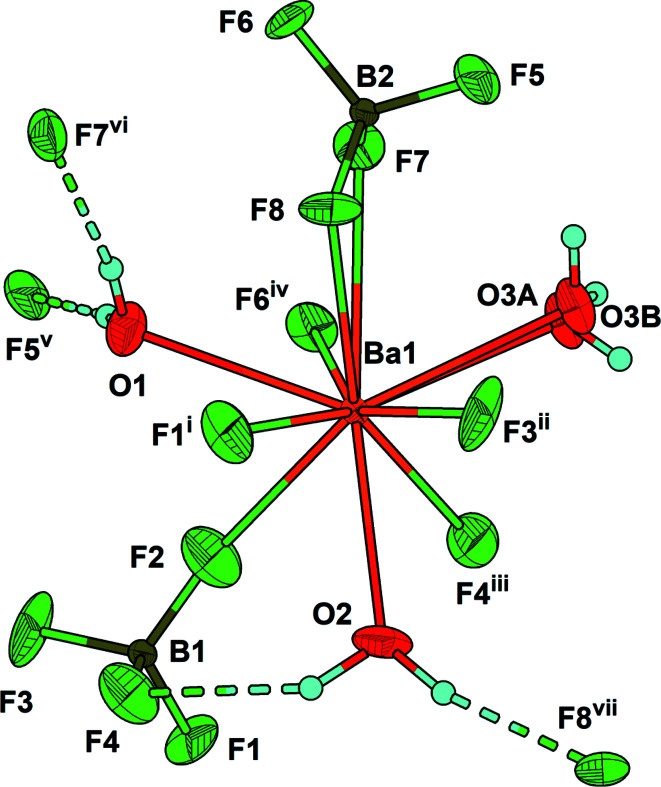
The environment of the Ba^2+^ cation in the crystal structure of LT-Ba(BF_4_)_2_(H_2_O)_3_. Displacement ellipsoids are drawn at the 50% probability level. Hydrogen bonds are shown as dashed lines. [Symmetry codes: (i) −*x* + 1, *y* − 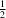 , −*z*; (ii) *x* + 1, *y*, *z*; (iii) −*x* + 1, *y* + 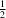 , −*z*; (iv) −*x* + 2, *y* + 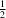 , −*z* + 1; (v) *x* − 1, *y*, *z*; (vi) −*x* + 2, *y* − 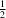 , −*z* + 1.]

The asymmetric unit of LT-Ba(BF_4_)_2_(H_2_O)_3_ contains one Ba^2+^ cation, two tetra­hedral BF_4_^−^ anions and three water mol­ecules, one of which (O3) is disordered over two sets of sites with approximately equal occupancy [ratio 0.56 (2):0.44 (2)]. The Ba^2+^ cation has a coordination number of 10 and is coordinated by seven F ligands from six BF_4_^−^ anions and by three water ligands (Fig. 1 ▸). In anhydrous Ba(BF_4_)_2_ (Bunič *et al.*, 2007 ▸), the Ba^2+^ cation is surrounded by ten BF_4_^−^ anions. The B(1)F_4_ unit in LT-Ba(BF_4_)_2_(H_2_O)_3_ is bound to four Ba^2+^ cations, while the B(2)F_4_ unit is connected in a chelate mode to one Ba^2+^ cation and to another *via* a *μ*_2_-bridging F ligand. Each [BaF_7_O_3_] coordination polyhedron shares two vertices with two other [BaF_7_O_3_] polyhedra. The shortest Ba⋯Ba distances amounts to 5.9210 (2) Å. Ba—F bond lengths range from 2.698 (7) to 3.035 (8) Å, and Ba—O bond lengths from 2.777 (9) to 2.821 (8) Å (for ordered water mol­ecules). The spread of Ba—F distances in LT-Ba(BF_4_)_2_(H_2_O)_3_ is greater than for the RT-modification [2.729 (4) to 2.843 (17) Å; Charkin *et al.*, 2023 ▸]. The B—F distances in LT-Ba(BF_4_)_2_(H_2_O)_3_ are in normal ranges, 1.352 (12)–1.406 (16) Å.

**Figure 2:**
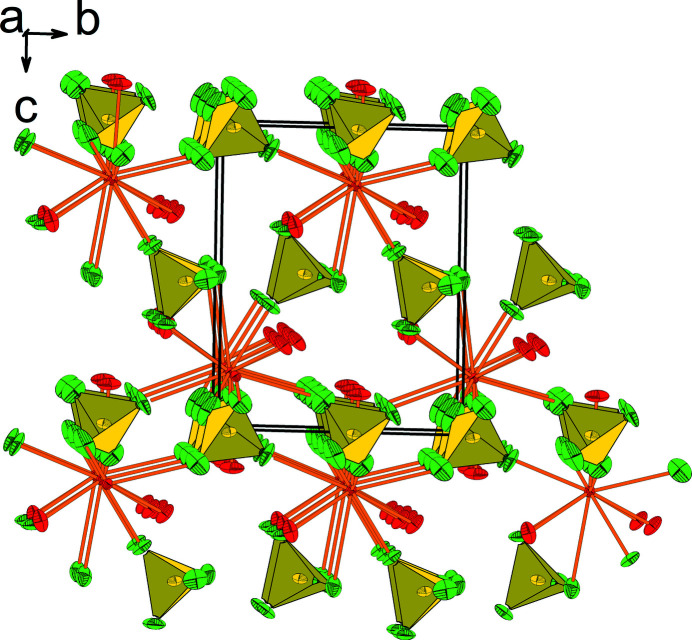
Crystal structure of LT-Ba(BF_4_)_2_(H_2_O)_3_ in a view approximately along [100]. Displacement ellipsoids are the same as in Fig. 1 ▸. Display of hydrogen-bonding inter­actions was omitted for clarity.

The packing of LT-Ba(BF_4_)_2_(H_2_O)_3_ is shown in Fig. 2 ▸. The two ordered water mol­ecules form O—H⋯F hydrogen bonds, and the disordered water mol­ecule forms both O—H⋯F and O—H⋯O hydrogen bonds (Fig. 1 ▸, Table 1 ▸).

The RT unit cell in space group *C*222_1_ with *a* = 7.1763 (6) Å, *b* = 18.052 (2) Å, *c* = 7.1631 (6) Å (Charkin *et al.*, 2023 ▸) is related to the LT m*P* unit cell in *P*2_1_ by the transformation **–a, –c, 1/2a + 1/2b**, suggesting a *translationengleiche* symmetry relationship of index 2 (Müller, 2013 ▸). Considering the significant difference in crystal density for both modifications (2.59 g cm-^3^ for the RT modification at 300 K, 2.63 g cm^−3^ for the LT modification at 280 K, and 2.59 g cm^−3^ at 150 K), it can be assumed that the formation of a structure with more effective packing is the driving force of the observed phase transition.

We also tried to determine the temperature of the phase transition. It is noteworthy that at 280 K the LT modification remains unchanged, with significantly enlarged unit-cell parameters (Table 2 ▸). At 300 K, an ortho­rhom­bic cell was indexed with 100% of all observed reflections and with similar lattice parameters as given by Charkin *et al.* (2023 ▸). Thus, we can conclude that the ordered o*C* ⇌ m*P* phase transition of Ba(BF_4_)_2_(H_2_O)_3_ (accompanied by twinning of the monoclinic LT phase) occurs between 280 and 300 K.

## Synthesis and crystallization

Single crystals of Ba(BF_4_)_2_(H_2_O)_3_ were grown from an aqueous solution of Ba(BF_4_)_2_. Barium carbonate was added in small portions to 40%_wt_ HBF_4_. After completion of the gaseous CO_2_ release, the solution was deca­nted from residual BaCO_3_. Evaporation of water at room temperature yielded small crystals of Ba(BF_4_)_2_(H_2_O)_3_. Note that an excess of HBF_4_ led to the formation of crystals of anhydrous Ba(BF_4_)_2_ in our experiments.

## Refinement

Crystal data, data collection and structure refinement details are summarized in Table 3 ▸. The obtained crystals suffer from racemic twinning and additionally show twinning by pseudo-merohedry at decreasing temperature. To avoid complicated refinement, many crystals were tested until a crystal with a Flack parameter (Flack, 1983 ▸) close to zero was found. The twin law corresponding to a 180° rotation around the [100] direction was determined, and the reflection array was indexed as a two-component twin with a negligible amount of non-indexed reflections. Because of the relatively small amount (BASF = 0.26) of the second domain, the final refinement was performed with a HKLF5-type file containing reflections from the first domain and overlapping reflections. Because of unstable refinement, EADP commands in *SHELXL* (Sheldrick, 2015 ▸) were applied to the pair of disordered O3 atoms and also to the pair of B atoms. Hydrogen atoms were placed on calculated positions and refined with AFIX 7 restrictions. One reflection with an error/e.s.d. ratio of 5.5 was omitted.

## Supplementary Material

Crystal structure: contains datablock(s) I. DOI: 10.1107/S2414314623004881/wm4190sup1.cif

Structure factors: contains datablock(s) I. DOI: 10.1107/S2414314623004881/wm4190Isup3.hkl

CCDC reference: 2267617

Additional supporting information:  crystallographic information; 3D view; checkCIF report

## Figures and Tables

**Table 1:** Hydrogen-bond geometry (Å, °)

*D*—H⋯*A*	*D*—H	H⋯*A*	*D*⋯*A*	*D*—H⋯*A*
O1—H1*A*⋯F5^i^	0.829 (10)	2.075 (8)	2.892 (12)	168.8 (7)
O1—H1*B*⋯F7^ii^	0.842 (10)	2.016 (7)	2.849 (13)	170.5 (7)
O2—H2*A*⋯F4	0.974 (9)	2.331 (9)	3.119 (13)	137.4 (5)
O2—H2*B*⋯F8^iii^	0.977 (9)	2.053 (11)	3.002 (15)	163.5 (7)
O3*A*—H3*AA*⋯O2^iii^	0.88 (2)	2.191 (11)	2.96 (3)	146.5 (18)
O3*A*—H3*AB*⋯F7^iv^	0.84 (2)	2.386 (8)	3.13 (2)	147.7 (17)
O3*B*—H3*BA*⋯F5	0.86 (3)	2.355 (9)	3.15 (3)	153.0 (16)

Symmetry codes: (i) 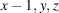 ; (ii) 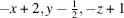 ; (iii) 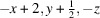 ; (iv) 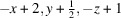 .

**Table 2:** Unit-cell parameters (Å, °, Å^3^) of Ba(BF_4_)_2_(H_2_O)_3_ at different temperatures (K)

*T*	*a*	*b*	*c*	*β*	*V*
100	7.0406 (5)	7.1567 (3)	9.3926 (9)	109.292 (7)	446.69 (5)
150	7.0550 (4)	7.1706 (3)	9.4182 (6)	109.295 (7)	449.68 (5)
280	7.1469 (5)	7.1775 (4)	9.5820 (7)	110.254 (6)	461.13 (5)
300	7.1763 (6)	18.052 (2)	7.1631 (6)		927.93 (15)

**Table 3:** Experimental details

Crystal data	
Chemical formula	Ba(BF_4_)_2_(H_2_O)_3_
*M*_r_	365.01
Crystal system, space group	Monoclinic, *P*2_1_
Temperature (K)	150
*a*, *b*, *c* (Å)	7.0550 (4), 7.1706 (3), 9.4182 (6)
β (°)	109.295 (7)
*V* (Å^3^)	449.68 (5)
*Z*	2
Radiation type	Mo *K*α
μ (mm^−1^)	4.53
Crystal size (mm)	0.36 × 0.27 × 0.07

Data collection	
Diffractometer	New Gemini, Dual, Cu at home/near, Atlas
Absorption correction	Analytical (*CrysAlis PRO*; Rigaku OD, 2023)
*T*_min_, *T*_max_	0.075, 0.472
No. of measured, independent and observed [*I* > 2σ(*I*)] reflections	2386, 2386, 2212
(sin θ/λ)_max_ (Å^−1^)	0.674

Refinement	
*R*[*F* ^2^ > 2σ(*F* ^2^)], *wR*(*F* ^2^), *S*	0.036, 0.089, 1.07
No. of reflections	2386
No. of parameters	120
No. of restraints	1
H-atom treatment	H-atom parameters constrained
Δρ_max_, Δρ_min_ (e Å^−3^)	0.93, −1.05
Absolute structure	Classical Flack method preferred over Parsons because s.u. lower
Absolute structure parameter	−0.03 (4)

Computer programs: *CrysAlis PRO* (Rigaku OD, 2023 ▸), *SHELXS* (Sheldrick, 2008 ▸), *SHELXL* (Sheldrick, 2015 ▸), *DIAMOND* (Putz *et al.*, 2023 ▸) and *OLEX2* (Dolomanov *et al.*, 2009 ▸).

## full crystallographic data

### Crystal data

**Table:**

Ba(BF_4_)_2_(H_2_O)_32_(BF_4_)·3(H_2_O)·Ba	*F*(000) = 336
*M**_r_* = 365.01	*D*_x_ = 2.696 Mg m^−^^3^
Monoclinic, *P*2_1_	Mo *K*α radiation, λ = 0.71073 Å
*a* = 7.0550 (4) Å	Cell parameters from 5692 reflections
*b* = 7.1706 (3) Å	θ = 3.0–28.2°
*c* = 9.4182 (6) Å	µ = 4.53 mm^−^^1^
β = 109.295 (7)°	*T* = 150 K
*V* = 449.68 (5) Å^3^	Plate, colourless
*Z* = 2	0.36 × 0.27 × 0.07 mm

### Data collection

**Table:**

New Gemini, Dual, Cu at home/near, Atlas diffractometer	2386 measured reflections
Radiation source: fine-focus sealed X-ray tube, Enhance (Mo) X-ray Source	2386 independent reflections
Graphite monochromator	2212 reflections with *I* > 2σ(*I*)
Detector resolution: 10.6426 pixels mm^-1^	θ_max_ = 28.6°, θ_min_ = 2.3°
ω scans	*h* = −9→9
Absorption correction: analytical (CrysAlisPro; Rigaku OD, 2023)	*k* = −9→9
*T*_min_ = 0.075, *T*_max_ = 0.472	*l* = −12→12

### Refinement

**Table:**

Refinement on *F*^2^	Hydrogen site location: difference Fourier map
Least-squares matrix: full	H-atom parameters constrained
*R*[*F*^2^ > 2σ(*F*^2^)] = 0.036	*w* = 1/[σ^2^(*F*_o_^2^) + (0.0656*P*)^2^] where *P* = (*F*_o_^2^ + 2*F*_c_^2^)/3
*wR*(*F*^2^) = 0.089	(Δ/σ)_max_ = 0.009
*S* = 1.07	Δρ_max_ = 0.93 e Å^−^^3^
2386 reflections	Δρ_min_ = −1.05 e Å^−^^3^
120 parameters	Absolute structure: Classical Flack method preferred over Parsons because s.u. lower
1 restraint	Absolute structure parameter: −0.03 (4)

### Special details

**Table:**

Geometry. All esds (except the esd in the dihedral angle between two l.s. planes) are estimated using the full covariance matrix. The cell esds are taken into account individually in the estimation of esds in distances, angles and torsion angles; correlations between esds in cell parameters are only used when they are defined by crystal symmetry. An approximate (isotropic) treatment of cell esds is used for estimating esds involving l.s. planes.
Refinement. Refined as a 2-component twin.1. Twinned data refinement Scales: 0.738 (3) 0.262 (3) 2. Fixed Uiso At 1.5 times of: All O(H,H,H,H,H,H,H,H) groups 3. Uiso/Uaniso restraints and constraints Uanis(O3B) = Uanis(O3A) Uanis(B2) = Uanis(B1) Uanis(F4) = Uanis(F2) 4. Others Sof(O3B)=Sof(H3BA)=Sof(H3BB)=1-FVAR(1) Sof(O3A)=Sof(H3AA)=Sof(H3AB)=FVAR(1) Fixed X: H1A(1.393391) H1B(1.25841) H3AA(0.82474) H3AB(0.919749) H3BA(0.70805) H3BB(0.82046) H2A(1.436299) H2B(1.27384) Fixed Y: H1A(0.843861) H1B(0.70964) H3AA(1.34448) H3AB(1.39917) H3BA(1.19412) H3BB(1.354321) H2A(0.999499) H2B(1.11445) Fixed Z: H1A(0.62203) H1B(0.62415) H3AA(0.77378) H3AB(0.663441) H3BA(0.651361) H3BB(0.66705) H2A(1.1758) H2B(1.21389)

### Fractional atomic coordinates and isotropic or equivalent isotropic displacement parameters (Å^2^)

**Table:**

	*x*	*y*	*z*	*U*_iso_*/*U*_eq_	Occ. (<1)
Ba1	0.84596 (6)	0.54566 (15)	0.18707 (5)	0.01069 (17)	
F1	0.1744 (12)	0.6957 (9)	−0.0772 (9)	0.0392 (19)	
F2	0.4423 (11)	0.5754 (14)	0.0977 (9)	0.0449 (16)	
F3	0.1320 (13)	0.4559 (13)	0.0610 (12)	0.050 (3)	
F4	0.2873 (13)	0.4098 (13)	−0.1086 (11)	0.0449 (16)	
F5	1.4219 (11)	0.4296 (12)	0.4972 (9)	0.0361 (18)	
F6	1.2726 (11)	0.2125 (11)	0.5958 (8)	0.0299 (16)	
F7	1.1150 (11)	0.4849 (9)	0.5097 (9)	0.0311 (18)	
F8	1.1510 (13)	0.2707 (18)	0.3442 (11)	0.035 (3)	
O1	0.6644 (13)	0.2926 (16)	0.3248 (12)	0.031 (2)	
O2	0.6901 (12)	0.565 (2)	−0.1296 (9)	0.033 (2)	
B1	0.2610 (14)	0.537 (4)	−0.0048 (10)	0.0136 (15)	
B2	1.2421 (17)	0.3491 (17)	0.4874 (13)	0.0136 (15)	
O3A	1.128 (3)	0.813 (4)	0.298 (2)	0.025 (4)	0.56 (2)
O3B	1.197 (4)	0.756 (4)	0.284 (3)	0.025 (4)	0.44 (2)
H1A	0.606609	0.343861	0.377970	0.038*	
H1B	0.741590	0.209640	0.375850	0.038*	
H3AA	1.175261	0.844480	0.226220	0.038*	0.56 (2)
H3AB	1.080250	0.899170	0.336559	0.038*	0.56 (2)
H3BA	1.291950	0.694120	0.348640	0.038*	0.44 (2)
H3BB	1.179539	0.854321	0.332950	0.038*	0.44 (2)
H2A	0.563701	0.499499	−0.175799	0.038*	
H2B	0.726160	0.614450	−0.213891	0.038*	

### Atomic displacement parameters (Å^2^)

**Table:**

	*U*^11^	*U*^22^	*U*^33^	*U*^12^	*U*^13^	*U*^23^
Ba1	0.0098 (2)	0.0107 (2)	0.0114 (3)	−0.0003 (4)	0.00317 (18)	−0.0011 (4)
F1	0.044 (5)	0.021 (3)	0.049 (5)	0.011 (3)	0.010 (4)	0.022 (3)
F2	0.029 (3)	0.052 (4)	0.052 (4)	0.000 (3)	0.011 (3)	−0.013 (3)
F3	0.043 (5)	0.048 (5)	0.082 (7)	0.010 (4)	0.051 (5)	0.030 (5)
F4	0.029 (3)	0.052 (4)	0.052 (4)	0.000 (3)	0.011 (3)	−0.013 (3)
F5	0.014 (4)	0.056 (5)	0.038 (5)	−0.005 (3)	0.008 (3)	0.003 (4)
F6	0.037 (4)	0.031 (4)	0.022 (4)	0.005 (3)	0.010 (3)	0.016 (3)
F7	0.028 (4)	0.024 (4)	0.040 (5)	0.008 (3)	0.009 (3)	0.001 (3)
F8	0.040 (6)	0.048 (6)	0.011 (4)	0.006 (4)	0.000 (3)	−0.002 (5)
O1	0.029 (5)	0.022 (5)	0.045 (7)	0.002 (3)	0.017 (5)	0.008 (4)
O2	0.026 (4)	0.055 (7)	0.013 (3)	0.006 (6)	0.000 (3)	−0.001 (6)
B1	0.010 (3)	0.021 (4)	0.010 (3)	−0.003 (4)	0.003 (3)	0.002 (4)
B2	0.010 (3)	0.021 (4)	0.010 (3)	−0.003 (4)	0.003 (3)	0.002 (4)
O3A	0.020 (10)	0.028 (11)	0.034 (8)	−0.007 (6)	0.017 (7)	−0.017 (7)
O3B	0.020 (10)	0.028 (11)	0.034 (8)	−0.007 (6)	0.017 (7)	−0.017 (7)

### Geometric parameters (Å, º)

**Table:**

Ba1—F1^i^	2.700 (6)	F4—B1	1.39 (2)
Ba1—F2	2.698 (7)	F5—B2	1.369 (13)
Ba1—F3^ii^	2.735 (7)	F6—B2	1.379 (14)
Ba1—F4^iii^	2.791 (9)	F7—B2	1.385 (14)
Ba1—F6^iv^	2.728 (7)	F8—B2	1.406 (16)
Ba1—F7	3.035 (8)	O1—H1A	0.83
Ba1—F8	2.933 (10)	O1—H1B	0.84
Ba1—O1	2.777 (9)	O2—H2A	0.97
Ba1—O2	2.821 (8)	O2—H2B	0.98
Ba1—O3A	2.71 (2)	O3A—H3AA	0.88
Ba1—O3B	2.78 (2)	O3A—H3AB	0.84
F1—B1	1.36 (2)	O3B—H3BA	0.86
F2—B1	1.352 (12)	O3B—H3BB	0.88
F3—B1	1.387 (15)		
			
F1^i^—Ba1—F3^ii^	64.4 (3)	O2—Ba1—F7	164.1 (2)
F1^i^—Ba1—F4^iii^	142.9 (3)	O2—Ba1—F8	122.1 (3)
F1^i^—Ba1—F6^iv^	134.8 (2)	O3A—Ba1—F3^ii^	77.3 (5)
F1^i^—Ba1—F7	100.8 (2)	O3A—Ba1—F4^iii^	65.1 (6)
F1^i^—Ba1—F8	60.4 (3)	O3A—Ba1—F6^iv^	76.6 (4)
F1^i^—Ba1—O1	66.3 (3)	O3A—Ba1—F7	65.2 (6)
F1^i^—Ba1—O2	71.8 (3)	O3A—Ba1—F8	87.7 (5)
F1^i^—Ba1—O3A	138.1 (5)	O3A—Ba1—O1	132.5 (5)
F1^i^—Ba1—O3B	123.8 (6)	O3A—Ba1—O2	110.6 (6)
F2—Ba1—F1^i^	92.1 (3)	O3B—Ba1—F7	63.6 (6)
F2—Ba1—F3^ii^	137.5 (3)	O3B—Ba1—F8	76.9 (6)
F2—Ba1—F4^iii^	67.4 (3)	O3B—Ba1—O2	108.2 (6)
F2—Ba1—F6^iv^	69.2 (2)	B1—F1—Ba1^iii^	157.8 (7)
F2—Ba1—F7	124.8 (2)	B1—F2—Ba1	149.2 (8)
F2—Ba1—F8	137.2 (3)	B1—F3—Ba1^v^	141.5 (12)
F2—Ba1—O1	66.1 (3)	B1—F4—Ba1^i^	133.4 (10)
F2—Ba1—O2	70.4 (2)	B2—F6—Ba1^vi^	148.7 (7)
F2—Ba1—O3A	128.9 (6)	B2—F7—Ba1	99.9 (6)
F2—Ba1—O3B	142.5 (6)	B2—F8—Ba1	104.0 (8)
F3^ii^—Ba1—F4^iii^	109.7 (3)	Ba1—O1—H1A	113.0
F3^ii^—Ba1—F7	95.4 (3)	Ba1—O1—H1B	115.0
F3^ii^—Ba1—F8	62.4 (3)	H1A—O1—H1B	109.0
F3^ii^—Ba1—O1	124.7 (3)	Ba1—O2—H2A	115.0
F3^ii^—Ba1—O2	68.8 (3)	F1—B1—F3	108.8 (10)
F3^ii^—Ba1—O3B	64.1 (6)	F1—B1—F4	109.7 (8)
F4^iii^—Ba1—F7	116.4 (2)	F2—B1—F1	110.5 (17)
F4^iii^—Ba1—F8	152.8 (3)	F2—B1—F3	111.8 (9)
F4^iii^—Ba1—O2	72.2 (4)	F2—B1—F4	108.7 (10)
F6^iv^—Ba1—F3^ii^	151.8 (3)	F3—B1—F4	107.3 (17)
F6^iv^—Ba1—F4^iii^	68.2 (3)	F5—B2—F6	109.6 (9)
F6^iv^—Ba1—F7	63.8 (2)	F5—B2—F7	109.0 (10)
F6^iv^—Ba1—F8	105.8 (3)	F5—B2—F8	110.6 (10)
F6^iv^—Ba1—O1	68.5 (3)	F6—B2—F7	109.8 (9)
F6^iv^—Ba1—O2	131.5 (3)	F6—B2—F8	109.7 (10)
F6^iv^—Ba1—O3B	88.8 (5)	F7—B2—F8	108.2 (10)
F8—Ba1—F7	44.5 (2)	Ba1—O3A—H3AA	108.0
O1—Ba1—F4^iii^	124.6 (3)	Ba1—O3A—H3AB	110.0
O1—Ba1—F7	70.8 (3)	H3AA—O3A—H3AB	117.0
O1—Ba1—F8	72.5 (3)	Ba1—O3B—H3BA	111.0
O1—Ba1—O2	116.6 (3)	Ba1—O3B—H3BB	110.0
O1—Ba1—O3B	134.4 (6)	H3BA—O3B—H3BB	104.0
			
Ba1^iii^—F1—B1—F2	25 (2)	Ba1^i^—F4—B1—F3	−3.8 (15)
Ba1^iii^—F1—B1—F3	148.0 (14)	Ba1^vi^—F6—B2—F5	136.5 (11)
Ba1^iii^—F1—B1—F4	−95 (2)	Ba1^vi^—F6—B2—F7	16.9 (19)
Ba1—F2—B1—F1	−111 (2)	Ba1^vi^—F6—B2—F8	−101.9 (14)
Ba1—F2—B1—F3	127.5 (12)	Ba1—F7—B2—F5	103.0 (8)
Ba1—F2—B1—F4	9 (3)	Ba1—F7—B2—F6	−137.0 (8)
Ba1^v^—F3—B1—F1	−31.2 (17)	Ba1—F7—B2—F8	−17.3 (9)
Ba1^v^—F3—B1—F2	91.1 (18)	Ba1—F8—B2—F5	−101.1 (9)
Ba1^v^—F3—B1—F4	−149.9 (11)	Ba1—F8—B2—F6	138.0 (7)
Ba1^i^—F4—B1—F1	−121.8 (12)	Ba1—F8—B2—F7	18.2 (10)
Ba1^i^—F4—B1—F2	117.3 (11)		

Symmetry codes: (i) −*x*+1, *y*−1/2, −*z*; (ii) *x*+1, *y*, *z*; (iii) −*x*+1, *y*+1/2, −*z*; (iv) −*x*+2, *y*+1/2, −*z*+1; (v) *x*−1, *y*, *z*; (vi) −*x*+2, *y*−1/2, −*z*+1.

### Hydrogen-bond geometry (Å, º)

**Table:**

*D*—H···*A*	*D*—H	H···*A*	*D*···*A*	*D*—H···*A*
O1—H1*A*···F5^v^	0.829 (10)	2.075 (8)	2.892 (12)	168.8 (7)
O1—H1*B*···F7^vi^	0.842 (10)	2.016 (7)	2.849 (13)	170.5 (7)
O2—H2*A*···F4	0.974 (9)	2.331 (9)	3.119 (13)	137.4 (5)
O2—H2*B*···F8^vii^	0.977 (9)	2.053 (11)	3.002 (15)	163.5 (7)
O3*A*—H3*AA*···O2^vii^	0.88 (2)	2.191 (11)	2.96 (3)	146.5 (18)
O3*A*—H3*AB*···F7^iv^	0.84 (2)	2.386 (8)	3.13 (2)	147.7 (17)
O3*B*—H3*BA*···F5	0.86 (3)	2.355 (9)	3.15 (3)	153.0 (16)

Symmetry codes: (iv) −*x*+2, *y*+1/2, −*z*+1; (v) *x*−1, *y*, *z*; (vi) −*x*+2, *y*−1/2, −*z*+1; (vii) −*x*+2, *y*+1/2, −*z*.

### Transformation matrix oC–mP (HT–LT phase transition of Ba(BF_4_)_2_(H_3_O)_3_

**Table:**

-1	0	0
0	0	-1
1/2	-1/2	0
